# (3*E*,5*E*)-3,5-Dibenzyl­idene-1-phenethyl­piperidin-4-one

**DOI:** 10.1107/S1600536811035744

**Published:** 2011-09-14

**Authors:** Mohamed Ashraf Ali, Tan Soo Choon, Abdulrahman I. Almansour, Tara Shahani, Hoong-Kun Fun

**Affiliations:** a Institute for Research in Molecular Medicine, Universiti Sains Malaysia, Minden 11800, Penang, Malaysia; b School of Physical Sciences, Universiti Sains Malaysia, Minden 11800, Penang, Malaysia; c Department of Chemistry, College of Sciences, King Saud University, PO Box 2455, Riyadh, 11451, Saudi Arabia; d X-ray Crystallography Unit, School of Physics, Universiti Sains Malaysia, 11800 USM, Penang, Malaysia

## Abstract

In the title compound, C_27_H_25_NO, the piperidine ring adopts an envelope conformation with the N atom at the flap position. The two benzylidene-benzene rings are oriented at a dihedral angle of 8.5 (1)°. In the crystal, the mol­ecules are linked into centrosymmetric dimers by pairs of inter­molecular C—H⋯O hydrogen bonds. The dimers are connected *via* C—H⋯π inter­actions involving the phenyl rings.

## Related literature

For the biological activity of piperidine compounds, see: Asano *et al.* (2000 ▶); Scriabine (1980 ▶); Watson *et al.* (2000 ▶); Risi (2008 ▶). For bond-length data, see: Allen *et al.* (1987 ▶). For ring puckering parameters, see: Cremer & Pople (1975 ▶).
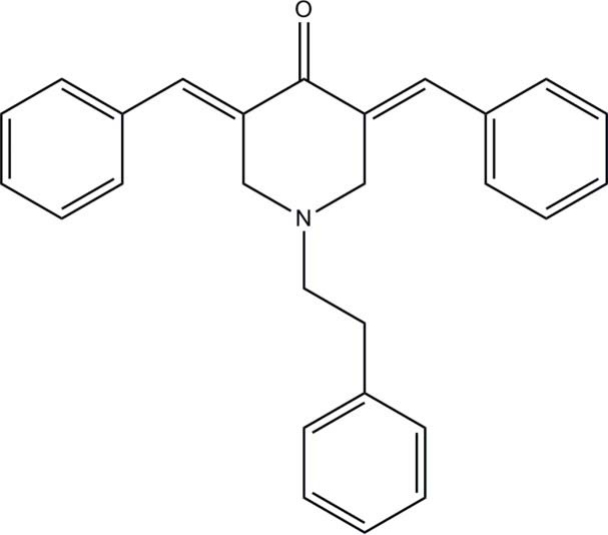


## Experimental

### 

#### Crystal data

C_27_H_25_NO
*M* *_r_* = 379.48
Monoclinic, 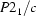

*a* = 11.4785 (2) Å
*b* = 5.8396 (1) Å
*c* = 30.9591 (5) Å
β = 106.412 (1)°
*V* = 1990.63 (6) Å^3^
*Z* = 4
Mo *K*α radiation
μ = 0.08 mm^−1^
*T* = 296 K
0.36 × 0.30 × 0.11 mm

#### Data collection

Bruker APEXII DUO CCD area-detector diffractometer
Absorption correction: multi-scan (*SADABS*; Bruker, 2009 ▶) *T*_min_ = 0.973, *T*_max_ = 0.992
21942 measured reflections
5877 independent reflections
4370 reflections with *I* > 2σ(*I*)
*R*_int_ = 0.048

#### Refinement

*R*[*F* ^2^ > 2σ(*F* ^2^)] = 0.070
*wR*(*F* ^2^) = 0.173
*S* = 1.08
5877 reflections
262 parameters
H-atom parameters constrained
Δρ_max_ = 0.40 e Å^−3^
Δρ_min_ = −0.28 e Å^−3^

### 

Data collection: *APEX2* (Bruker, 2009 ▶); cell refinement: *SAINT* (Bruker, 2009 ▶); data reduction: *SAINT*; program(s) used to solve structure: *SHELXTL* (Sheldrick, 2008 ▶); program(s) used to refine structure: *SHELXTL*; molecular graphics: *SHELXTL*; software used to prepare material for publication: *SHELXTL* and *PLATON* (Spek, 2009 ▶).

## Supplementary Material

Crystal structure: contains datablock(s) global, I. DOI: 10.1107/S1600536811035744/ci5200sup1.cif

Structure factors: contains datablock(s) I. DOI: 10.1107/S1600536811035744/ci5200Isup2.hkl

Supplementary material file. DOI: 10.1107/S1600536811035744/ci5200Isup3.cml

Additional supplementary materials:  crystallographic information; 3D view; checkCIF report

## Figures and Tables

**Table 1:** Hydrogen-bond geometry (Å, °) *Cg*1, *Cg*2 and *Cg*3 are centroids of the C1–C6, C14–C19 and C22–C27 phenyl rings, respectively.

*D*—H⋯*A*	*D*—H	H⋯*A*	*D*⋯*A*	*D*—H⋯*A*
C13—H13*A*⋯O1^i^	0.95	2.54	3.425 (3)	155
C15—H15*A*⋯O1^i^	0.95	2.49	3.345 (3)	150
C2—H2*A*⋯*Cg*3^ii^	0.95	2.88	3.579 (2)	131
C23—H23*A*⋯*Cg*1^iii^	0.95	2.99	3.640 (2)	127
C26—H26*A*⋯*Cg*2^iv^	0.95	2.89	3.556 (2)	128

Symmetry codes: (i) 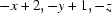; (ii) 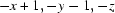; (iii) 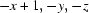; (iv) 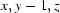.

**Fig. 1.**
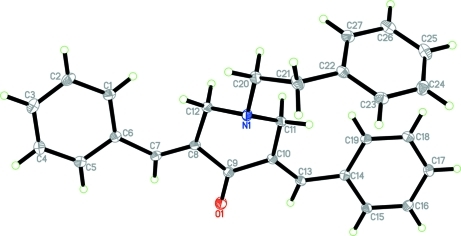
The molecular structure of the title compound, showing 30% probability displacement ellipsoids.

**Fig. 2.**
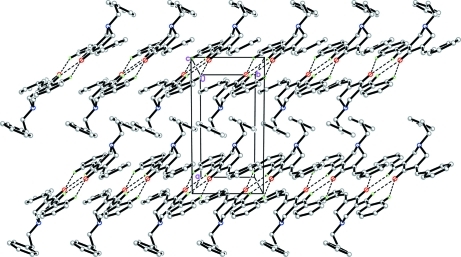
The crystal packing of the title compound. Intermolecular hydrogen bonds are shown as dashed lines.

## supplementary crystallographic information

### Comment

Piperidines are very important compounds because of their presence in numerous alkaloids, pharmaceuticals, agrochemical and as synthetic intermediates. Biologically active alkaloids of the substituted piperidine ring system have been targeted for their total or partial synthesis. During a fairly recent 10-year period, several thousand piperidine compounds have been mentioned in clinical and preclinical studies (Watson *et al.*, 2000). Selective inhibition of a number of enzymes involved in the binding and processing of glycoproteins has rendered piperidine alkaloids as important tools in the study of biochemical pathways (Asano *et al.*, 2000). Piperidine derivatives are found to possess pharmacological activity and form an essential part of the molecular structures of important drugs such as raloxifene and minoxidil (Risi, 2008). A new neuroleptics has found that the piperidine derivatives have high affinity for *CNS* (Scriabine, 1980).

In the title compound (Fig. 1), the piperidine (N1/C8–C12) ring is attached to three benzene (C1–C6), (C14–C19) and (C22–C27) rings via butane (C6–C8) and prop-1-ene (C20–C22) groups. The piperidine ring adopts an envelope conformation (Cremer & Pople, 1975) with puckering parameters of Q = 0.556 (2) Å, Θ = 60.3 (2)° and φ = 357.5 (2)°. Atom N1 deviates from the C8-C12 plane by 0.738 (2) Å. The two benzyl phenyl rings are oriented at a dihedral angle of 8.5 (1)°. The bond lengths (Allen *et al.*,1987) and angles are within normal ranges.

In the crystal structure (Fig. 2), intermolecular C15—H15A···O1 and C13—H13A···O1 hydrogen bonds link the molecules into centrosymmetric dimers each containing two *R*^1^_2_(6) ring motifs. In addition, the crystal structure is stabilized by C—H···π interactions (Table 1).

### Experimental

A mixture of 1-phenethyl-4-piperonidone (0.001 mmol) and benzaldehyde (0.002 mmol) were dissolved in methanol (10 ml) and 30% sodium hydroxide solution (5 ml) was added. The mixture was stirred for 5 h. After completion of the reaction as evident from TLC, the mixture was poured into crushed ice and then was neutralized with conentrated HCl. The precipitated solid was filtered, washed with water and recrystallized from ethanol to obtain the title compound as light yellow crystals.

### Refinement

H atoms were positioned geometrically [C–H = 0.95 or 0.99 Å] and refined using a riding model, with *U*_iso_(H) = 1.2 *U*_eq_(C).

### Crystal data

**Table:**

C_27_H_25_NO	*F*(000) = 808
*M**_r_* = 379.48	*D*_x_ = 1.266 Mg m^−^^3^
Monoclinic, *P*2_1_/*c*	Mo *K*α radiation, λ = 0.71073 Å
Hall symbol: -P 2ybc	Cell parameters from 5149 reflections
*a* = 11.4785 (2) Å	θ = 2.7–30.1°
*b* = 5.8396 (1) Å	µ = 0.08 mm^−^^1^
*c* = 30.9591 (5) Å	*T* = 296 K
β = 106.412 (1)°	Plate, light yellow
*V* = 1990.63 (6) Å^3^	0.36 × 0.30 × 0.11 mm
*Z* = 4	

### Data collection

**Table:**

Bruker APEXII DUO CCD area-detector diffractometer	5877 independent reflections
Radiation source: fine-focus sealed tube	4370 reflections with *I* > 2σ(*I*)
graphite	*R*_int_ = 0.048
φ and ω scans	θ_max_ = 30.2°, θ_min_ = 1.9°
Absorption correction: multi-scan (*SADABS*; Bruker, 2009)	*h* = −16→16
*T*_min_ = 0.973, *T*_max_ = 0.992	*k* = −8→8
21942 measured reflections	*l* = −43→43

### Refinement

**Table:**

Refinement on *F*^2^	Primary atom site location: structure-invariant direct methods
Least-squares matrix: full	Secondary atom site location: difference Fourier map
*R*[*F*^2^ > 2σ(*F*^2^)] = 0.070	Hydrogen site location: inferred from neighbouring sites
*wR*(*F*^2^) = 0.173	H-atom parameters constrained
*S* = 1.08	*w* = 1/[σ^2^(*F*_o_^2^) + (0.0585*P*)^2^ + 1.7928*P*] where *P* = (*F*_o_^2^ + 2*F*_c_^2^)/3
5877 reflections	(Δ/σ)_max_ = 0.001
262 parameters	Δρ_max_ = 0.40 e Å^−^^3^
0 restraints	Δρ_min_ = −0.28 e Å^−^^3^

### Special details

**Table:**

Geometry. All e.s.d.'s (except the e.s.d. in the dihedral angle between two l.s. planes) are estimated using the full covariance matrix. The cell e.s.d.'s are taken into account individually in the estimation of e.s.d.'s in distances, angles and torsion angles; correlations between e.s.d.'s in cell parameters are only used when they are defined by crystal symmetry. An approximate (isotropic) treatment of cell e.s.d.'s is used for estimating e.s.d.'s involving l.s. planes.
Refinement. Refinement of *F*^2^ against ALL reflections. The weighted *R*-factor *wR* and goodness of fit *S* are based on *F*^2^, conventional *R*-factors *R* are based on *F*, with *F* set to zero for negative *F*^2^. The threshold expression of *F*^2^ > σ(*F*^2^) is used only for calculating *R*-factors(gt) *etc*. and is not relevant to the choice of reflections for refinement. *R*-factors based on *F*^2^ are statistically about twice as large as those based on *F*, and *R*- factors based on ALL data will be even larger.

### Fractional atomic coordinates and isotropic or equivalent isotropic displacement parameters (Å^2^)

**Table:**

	*x*	*y*	*z*	*U*_iso_*/*U*_eq_	
O1	0.94565 (15)	0.2948 (3)	0.03565 (5)	0.0356 (4)	
N1	0.66007 (14)	−0.1134 (3)	−0.01094 (5)	0.0206 (3)	
C1	0.82777 (17)	−0.3600 (4)	0.13159 (7)	0.0236 (4)	
H1A	0.8069	−0.4400	0.1037	0.028*	
C2	0.80770 (18)	−0.4622 (4)	0.16937 (7)	0.0279 (4)	
H2A	0.7710	−0.6092	0.1670	0.033*	
C3	0.84119 (18)	−0.3500 (4)	0.21060 (7)	0.0293 (5)	
H3A	0.8261	−0.4188	0.2363	0.035*	
C4	0.89693 (18)	−0.1369 (4)	0.21413 (7)	0.0272 (4)	
H4A	0.9221	−0.0614	0.2424	0.033*	
C5	0.91570 (17)	−0.0350 (4)	0.17640 (6)	0.0235 (4)	
H5A	0.9548	0.1099	0.1793	0.028*	
C6	0.87861 (16)	−0.1397 (3)	0.13407 (6)	0.0204 (4)	
C7	0.89514 (16)	−0.0098 (3)	0.09573 (6)	0.0205 (4)	
H7A	0.9543	0.1083	0.1035	0.025*	
C8	0.84016 (16)	−0.0302 (3)	0.05124 (6)	0.0196 (4)	
C9	0.87531 (17)	0.1378 (4)	0.02060 (6)	0.0222 (4)	
C10	0.82163 (16)	0.1054 (3)	−0.02901 (6)	0.0195 (4)	
C11	0.72482 (17)	−0.0742 (3)	−0.04479 (6)	0.0212 (4)	
H11A	0.6666	−0.0240	−0.0733	0.025*	
H11B	0.7626	−0.2191	−0.0506	0.025*	
C12	0.74518 (17)	−0.2049 (3)	0.03011 (6)	0.0210 (4)	
H12A	0.7849	−0.3440	0.0226	0.025*	
H12B	0.7001	−0.2486	0.0518	0.025*	
C13	0.86363 (16)	0.2420 (3)	−0.05619 (6)	0.0207 (4)	
H13A	0.9225	0.3511	−0.0411	0.025*	
C14	0.83292 (16)	0.2494 (3)	−0.10568 (6)	0.0196 (4)	
C15	0.86569 (17)	0.4483 (4)	−0.12484 (6)	0.0215 (4)	
H15A	0.9045	0.5699	−0.1058	0.026*	
C16	0.84249 (17)	0.4707 (4)	−0.17115 (6)	0.0236 (4)	
H16A	0.8645	0.6075	−0.1835	0.028*	
C17	0.78731 (17)	0.2938 (4)	−0.19943 (6)	0.0236 (4)	
H17A	0.7717	0.3088	−0.2311	0.028*	
C18	0.75506 (17)	0.0949 (4)	−0.18125 (7)	0.0238 (4)	
H18A	0.7174	−0.0266	−0.2006	0.029*	
C19	0.77752 (16)	0.0719 (3)	−0.13486 (6)	0.0213 (4)	
H19A	0.7551	−0.0654	−0.1228	0.026*	
C20	0.55955 (17)	−0.2752 (4)	−0.02625 (6)	0.0257 (4)	
H20A	0.5255	−0.3081	−0.0009	0.031*	
H20B	0.5918	−0.4207	−0.0346	0.031*	
C21	0.45702 (17)	−0.1920 (4)	−0.06630 (6)	0.0279 (5)	
H21A	0.3815	−0.2703	−0.0651	0.034*	
H21B	0.4454	−0.0264	−0.0620	0.034*	
C22	0.47052 (16)	−0.2249 (4)	−0.11326 (6)	0.0219 (4)	
C23	0.42776 (17)	−0.0546 (4)	−0.14575 (7)	0.0250 (4)	
H23A	0.3951	0.0829	−0.1376	0.030*	
C24	0.43255 (18)	−0.0842 (4)	−0.18977 (7)	0.0301 (5)	
H24A	0.4027	0.0320	−0.2116	0.036*	
C25	0.48105 (19)	−0.2839 (4)	−0.20181 (7)	0.0321 (5)	
H25A	0.4844	−0.3046	−0.2319	0.039*	
C26	0.52451 (18)	−0.4527 (4)	−0.17005 (7)	0.0297 (5)	
H26A	0.5584	−0.5888	−0.1782	0.036*	
C27	0.51871 (17)	−0.4237 (4)	−0.12612 (7)	0.0254 (4)	
H27A	0.5481	−0.5413	−0.1046	0.031*	

### Atomic displacement parameters (Å^2^)

**Table:**

	*U*^11^	*U*^22^	*U*^33^	*U*^12^	*U*^13^	*U*^23^
O1	0.0454 (9)	0.0320 (9)	0.0238 (7)	−0.0209 (8)	0.0005 (6)	0.0003 (7)
N1	0.0206 (7)	0.0229 (8)	0.0171 (7)	−0.0057 (7)	0.0032 (6)	0.0018 (6)
C1	0.0203 (8)	0.0207 (10)	0.0258 (9)	0.0000 (8)	−0.0002 (7)	0.0029 (8)
C2	0.0223 (9)	0.0249 (10)	0.0328 (10)	−0.0009 (8)	0.0017 (8)	0.0097 (9)
C3	0.0247 (9)	0.0348 (12)	0.0289 (10)	0.0011 (9)	0.0083 (8)	0.0088 (9)
C4	0.0256 (9)	0.0329 (12)	0.0231 (9)	0.0019 (9)	0.0068 (8)	0.0004 (9)
C5	0.0209 (8)	0.0227 (10)	0.0261 (9)	0.0000 (8)	0.0053 (7)	−0.0011 (8)
C6	0.0164 (8)	0.0202 (9)	0.0230 (9)	0.0015 (7)	0.0030 (7)	0.0027 (8)
C7	0.0203 (8)	0.0175 (9)	0.0230 (9)	−0.0006 (7)	0.0050 (7)	0.0022 (7)
C8	0.0202 (8)	0.0152 (9)	0.0222 (8)	−0.0015 (7)	0.0038 (7)	0.0004 (7)
C9	0.0221 (8)	0.0213 (10)	0.0210 (9)	−0.0039 (8)	0.0027 (7)	−0.0001 (8)
C10	0.0189 (8)	0.0195 (9)	0.0190 (8)	−0.0022 (7)	0.0034 (6)	−0.0019 (7)
C11	0.0213 (8)	0.0213 (10)	0.0206 (8)	−0.0056 (7)	0.0054 (7)	−0.0017 (7)
C12	0.0222 (8)	0.0197 (9)	0.0195 (8)	−0.0019 (7)	0.0032 (7)	0.0004 (7)
C13	0.0204 (8)	0.0197 (9)	0.0210 (8)	−0.0035 (7)	0.0039 (7)	−0.0024 (7)
C14	0.0169 (8)	0.0211 (9)	0.0215 (8)	−0.0014 (7)	0.0066 (6)	0.0001 (7)
C15	0.0212 (8)	0.0208 (9)	0.0226 (9)	−0.0043 (7)	0.0061 (7)	−0.0012 (8)
C16	0.0240 (9)	0.0224 (10)	0.0253 (9)	−0.0009 (8)	0.0085 (7)	0.0027 (8)
C17	0.0218 (8)	0.0286 (11)	0.0212 (8)	0.0009 (8)	0.0076 (7)	−0.0009 (8)
C18	0.0235 (9)	0.0231 (10)	0.0258 (9)	−0.0011 (8)	0.0082 (7)	−0.0052 (8)
C19	0.0221 (8)	0.0186 (9)	0.0246 (9)	−0.0023 (7)	0.0089 (7)	−0.0013 (8)
C20	0.0242 (9)	0.0311 (11)	0.0215 (9)	−0.0114 (9)	0.0056 (7)	−0.0004 (8)
C21	0.0216 (9)	0.0379 (12)	0.0242 (9)	−0.0059 (9)	0.0062 (7)	−0.0072 (9)
C22	0.0156 (8)	0.0251 (10)	0.0232 (9)	−0.0034 (7)	0.0026 (7)	−0.0033 (8)
C23	0.0189 (8)	0.0236 (10)	0.0309 (10)	−0.0015 (8)	0.0045 (7)	−0.0038 (8)
C24	0.0257 (9)	0.0349 (13)	0.0272 (10)	−0.0036 (9)	0.0034 (8)	0.0050 (9)
C25	0.0258 (10)	0.0464 (14)	0.0246 (10)	−0.0071 (10)	0.0079 (8)	−0.0083 (10)
C26	0.0216 (9)	0.0324 (12)	0.0347 (11)	−0.0006 (9)	0.0073 (8)	−0.0113 (10)
C27	0.0204 (8)	0.0242 (10)	0.0287 (10)	0.0007 (8)	0.0022 (7)	−0.0020 (8)

### Geometric parameters (Å, °)

**Table:**

O1—C9	1.223 (2)	C14—C15	1.403 (3)
N1—C20	1.463 (2)	C14—C19	1.404 (3)
N1—C11	1.464 (2)	C15—C16	1.388 (3)
N1—C12	1.467 (2)	C15—H15A	0.95
C1—C2	1.389 (3)	C16—C17	1.386 (3)
C1—C6	1.406 (3)	C16—H16A	0.95
C1—H1A	0.95	C17—C18	1.386 (3)
C2—C3	1.389 (3)	C17—H17A	0.95
C2—H2A	0.95	C18—C19	1.392 (3)
C3—C4	1.390 (3)	C18—H18A	0.95
C3—H3A	0.95	C19—H19A	0.95
C4—C5	1.381 (3)	C20—C21	1.528 (3)
C4—H4A	0.95	C20—H20A	0.99
C5—C6	1.399 (3)	C20—H20B	0.99
C5—H5A	0.95	C21—C22	1.517 (3)
C6—C7	1.466 (3)	C21—H21A	0.99
C7—C8	1.348 (2)	C21—H21B	0.99
C7—H7A	0.95	C22—C27	1.392 (3)
C8—C9	1.497 (3)	C22—C23	1.401 (3)
C8—C12	1.501 (3)	C23—C24	1.390 (3)
C9—C10	1.497 (3)	C23—H23A	0.95
C10—C13	1.344 (3)	C24—C25	1.388 (3)
C10—C11	1.506 (3)	C24—H24A	0.95
C11—H11A	0.99	C25—C26	1.382 (3)
C11—H11B	0.99	C25—H25A	0.95
C12—H12A	0.99	C26—C27	1.391 (3)
C12—H12B	0.99	C26—H26A	0.95
C13—C14	1.473 (3)	C27—H27A	0.95
C13—H13A	0.95		
			
C20—N1—C11	112.49 (15)	C15—C14—C19	117.78 (17)
C20—N1—C12	108.47 (15)	C15—C14—C13	116.76 (17)
C11—N1—C12	109.18 (14)	C19—C14—C13	125.42 (18)
C2—C1—C6	120.81 (19)	C16—C15—C14	121.22 (18)
C2—C1—H1A	119.6	C16—C15—H15A	119.4
C6—C1—H1A	119.6	C14—C15—H15A	119.4
C3—C2—C1	120.2 (2)	C17—C16—C15	120.15 (19)
C3—C2—H2A	119.9	C17—C16—H16A	119.9
C1—C2—H2A	119.9	C15—C16—H16A	119.9
C2—C3—C4	119.76 (19)	C18—C17—C16	119.66 (18)
C2—C3—H3A	120.1	C18—C17—H17A	120.2
C4—C3—H3A	120.1	C16—C17—H17A	120.2
C5—C4—C3	119.9 (2)	C17—C18—C19	120.45 (19)
C5—C4—H4A	120.1	C17—C18—H18A	119.8
C3—C4—H4A	120.1	C19—C18—H18A	119.8
C4—C5—C6	121.7 (2)	C18—C19—C14	120.73 (18)
C4—C5—H5A	119.2	C18—C19—H19A	119.6
C6—C5—H5A	119.2	C14—C19—H19A	119.6
C5—C6—C1	117.59 (18)	N1—C20—C21	114.45 (18)
C5—C6—C7	117.23 (18)	N1—C20—H20A	108.6
C1—C6—C7	125.18 (18)	C21—C20—H20A	108.6
C8—C7—C6	130.63 (18)	N1—C20—H20B	108.6
C8—C7—H7A	114.7	C21—C20—H20B	108.6
C6—C7—H7A	114.7	H20A—C20—H20B	107.6
C7—C8—C9	117.20 (17)	C22—C21—C20	118.26 (17)
C7—C8—C12	125.30 (17)	C22—C21—H21A	107.7
C9—C8—C12	117.49 (16)	C20—C21—H21A	107.7
O1—C9—C10	121.46 (17)	C22—C21—H21B	107.7
O1—C9—C8	121.10 (17)	C20—C21—H21B	107.7
C10—C9—C8	117.44 (16)	H21A—C21—H21B	107.1
C13—C10—C9	116.80 (17)	C27—C22—C23	118.27 (18)
C13—C10—C11	124.95 (17)	C27—C22—C21	122.41 (19)
C9—C10—C11	118.25 (16)	C23—C22—C21	119.24 (19)
N1—C11—C10	110.74 (15)	C24—C23—C22	120.8 (2)
N1—C11—H11A	109.5	C24—C23—H23A	119.6
C10—C11—H11A	109.5	C22—C23—H23A	119.6
N1—C11—H11B	109.5	C25—C24—C23	119.9 (2)
C10—C11—H11B	109.5	C25—C24—H24A	120.1
H11A—C11—H11B	108.1	C23—C24—H24A	120.1
N1—C12—C8	110.72 (16)	C26—C25—C24	119.97 (19)
N1—C12—H12A	109.5	C26—C25—H25A	120.0
C8—C12—H12A	109.5	C24—C25—H25A	120.0
N1—C12—H12B	109.5	C25—C26—C27	120.1 (2)
C8—C12—H12B	109.5	C25—C26—H26A	119.9
H12A—C12—H12B	108.1	C27—C26—H26A	119.9
C10—C13—C14	130.25 (18)	C26—C27—C22	120.9 (2)
C10—C13—H13A	114.9	C26—C27—H27A	119.5
C14—C13—H13A	114.9	C22—C27—H27A	119.5
			
C6—C1—C2—C3	−1.9 (3)	C9—C8—C12—N1	−30.1 (2)
C1—C2—C3—C4	−1.2 (3)	C9—C10—C13—C14	178.07 (19)
C2—C3—C4—C5	1.8 (3)	C11—C10—C13—C14	−2.5 (3)
C3—C4—C5—C6	0.8 (3)	C10—C13—C14—C15	163.0 (2)
C4—C5—C6—C1	−3.8 (3)	C10—C13—C14—C19	−19.2 (3)
C4—C5—C6—C7	175.79 (18)	C19—C14—C15—C16	1.0 (3)
C2—C1—C6—C5	4.3 (3)	C13—C14—C15—C16	178.98 (17)
C2—C1—C6—C7	−175.19 (18)	C14—C15—C16—C17	−0.8 (3)
C5—C6—C7—C8	−158.6 (2)	C15—C16—C17—C18	0.2 (3)
C1—C6—C7—C8	20.9 (3)	C16—C17—C18—C19	0.1 (3)
C6—C7—C8—C9	177.81 (19)	C17—C18—C19—C14	0.1 (3)
C6—C7—C8—C12	−1.1 (3)	C15—C14—C19—C18	−0.6 (3)
C7—C8—C9—O1	−4.1 (3)	C13—C14—C19—C18	−178.45 (18)
C12—C8—C9—O1	174.81 (19)	C11—N1—C20—C21	−64.8 (2)
C7—C8—C9—C10	175.55 (17)	C12—N1—C20—C21	174.35 (16)
C12—C8—C9—C10	−5.5 (3)	N1—C20—C21—C22	83.7 (2)
O1—C9—C10—C13	6.6 (3)	C20—C21—C22—C27	40.6 (3)
C8—C9—C10—C13	−173.14 (17)	C20—C21—C22—C23	−142.6 (2)
O1—C9—C10—C11	−172.94 (19)	C27—C22—C23—C24	0.5 (3)
C8—C9—C10—C11	7.4 (3)	C21—C22—C23—C24	−176.47 (18)
C20—N1—C11—C10	175.99 (16)	C22—C23—C24—C25	−0.5 (3)
C12—N1—C11—C10	−63.6 (2)	C23—C24—C25—C26	0.0 (3)
C13—C10—C11—N1	−152.95 (19)	C24—C25—C26—C27	0.6 (3)
C9—C10—C11—N1	26.5 (2)	C25—C26—C27—C22	−0.6 (3)
C20—N1—C12—C8	−171.40 (16)	C23—C22—C27—C26	0.1 (3)
C11—N1—C12—C8	65.7 (2)	C21—C22—C27—C26	176.92 (18)
C7—C8—C12—N1	148.79 (19)		

### Hydrogen-bond geometry (Å, °)

**Table:**

Cg1, Cg2 and Cg3 are centroids of the C1–C6, C14–C19 and C22–C27 phenyl rings, respectively.

**Table:**

*D*—H···*A*	*D*—H	H···*A*	*D*···*A*	*D*—H···*A*
C13—H13A···O1^i^	0.95	2.54	3.425 (3)	155
C15—H15A···O1^i^	0.95	2.49	3.345 (3)	150
C2—H2A···Cg3^ii^	0.95	2.88	3.579 (2)	131
C23—H23A···Cg1^iii^	0.95	2.99	3.640 (2)	127
C26—H26A···Cg2^iv^	0.95	2.89	3.556 (2)	128

Symmetry codes: (i) −*x*+2, −*y*+1, −*z*; (ii) −*x*+1, −*y*−1, −*z*; (iii) −*x*+1, −*y*, −*z*; (iv) *x*, *y*−1, *z*.
